# The influence of teacher care on middle school students’ social–emotional competence: evidence from the China Education Panel Survey (2013–2014)

**DOI:** 10.3389/fpsyg.2026.1748385

**Published:** 2026-02-27

**Authors:** Zhen Zhang, Xiangyan Li, Guo Juan, Chunhui Qi

**Affiliations:** 1 Faculty of Education, Henan Normal University, Xinxiang, China; 2 Faculty of Education, Henan University, Kaifeng, China

**Keywords:** teacher care, social-emotional competence, teacher-student relationship, migration status, middle school students

## Abstract

**Introduction:**

Social-Emotional Competence is regarded as a core ability necessary for individual development in the 21st century, playing a significant role in improving students’ academic performance and promoting social development. As key guides to student development, teachers play an important role in cultivating children’s social-emotional competence. This study aims to explore the impact of teacher care on students’ social-emotional competence and its underlying mechanism.

**Methods:**

A moderated mediation model was constructed based on the 2013-2014 data from the China Education Panel Survey (CEPS), the most recent nationally representative dataset available for this type of analysis. Using data on teacher care, teacher-student relationship, social-emotional competence, migration status, and relevant covariates, a total of 12,319 valid samples were analyzed through descriptive statistics, correlation analysis, mediation effect test, and moderated mediation analysis.

**Results:**

Teacher care positively predicted social-emotional competence, teacher-student relationship partially mediated the relationship between teacher care and social-emotional competence. Analysis of simple moderation effects suggests that students’ migration status moderated the relationship between teacher care and teacher-student relationship.

**Discussion:**

These findings highlight the critical role of enhancing teachers’ caring behaviors and fostering positive teacher-student relationships in improving adolescents’ social-emotional competence. This study holds significant practical implications for promoting the harmonious physical and mental health development of middle school students.

## 1 Introduction

In recent years, driven by the overall trajectory of international social development and a new wave of technological revolution, social–emotional competence (SEC) have been widely recognized as one of the core competencies students need to meet the challenges of the 21st century (Nanda et al., 2025). Researchers generally agree that SEC constitute a set of essential abilities developed and acquired by individuals in complex situations. These abilities are closely related to self-adaptation and social development (Du and Mao, 2019; Hassani, 2024). Against this backdrop, cultivating students’ SEC to better prepare them for future societies has become a central focus of international organizations and national educational systems, and has gradually entered the policy agendas of various countries (Müller et al., 2020; Steponavičius et al., 2023).

The concept of SEC was first introduced in 1994 by the Collaborative for Academic, Social, and Emotional Learning (CASEL). It is defined as the ability of individuals to navigate their emotions, build positive relationships with others, and make responsible decisions to address various challenges in social life during interactions with society (Collaborative for Academic, Social, and Emotional Learning [CASEL], 2020; Atkins et al., 2023). Adolescence is a critical developmental window for cultivating SEC (Purna et al., 2024). A substantial body of research has shown that adolescents’ academic achievement, mental health, and social development are closely related to their level of this competence (Olhaberry and Sieverson, 2022). Strong SEC not only facilitates the formation of positive interpersonal relationships among secondary school students (Papadopoulos, 2020; Lin et al., 2024), but also contributes to improved academic outcomes (Kim and Shin, 2021; Urruticoechea et al., 2025). Furthermore, it helps adolescents regulate negative emotions, reduce problem behaviors (Chen et al., 2025), and develop positive personality traits and sound character, thereby laying a solid foundation for future success (Lv et al., 2025). Conversely, low levels of SEC are associated with social difficulties, behavioral problems (Martinsone et al., 2022), and poorer mental health (Zhou et al., 2025), and even increase the risk of being bullied at school (Li et al., 2025). Therefore, it is of great significance to further explore the influencing factors and formation mechanisms of SEC.

According to Bronfenbrenner’s ecological systems theory, the development of an individual’s SEC is embedded within microsystems composed of the family, school, and peers (Bronfenbrenner, 1994; Main et al., 2025). Among these, teachers constitute a key group influencing students’ development within the school microsystem (Orr and Lavy, 2024). In educational practice, teacher care is an important dimension of teacher-student interactions and directly affects teachers’ ability to build supportive systems for students (Zhang et al., 2025a). Consequently, it enhances students’ learning engagement, self-confidence, well-being, sense of recognition, and mental health (Yin et al., 2023). Teachers’ supportive care further enables students to perceive a positive classroom collaborative atmosphere. Within such a constructive classroom environment, harmonious teacher-student relationships and peer friendships can be fostered, and students can develop a strong sense of belonging to their class and school. All these elements are closely associated with students’ SEC (Yuan et al., 2021). Studies have shown that teacher care and emotional support are essential in establishing positive teacher-student relationship, which are regarded as a foundational element in the development of adolescents’ SEC (Huang and Zeng, 2023). However, how teacher care specifically affects students’ SEC, and particularly the role that the teacher-student relationship plays in this process, remains insufficiently explored.

In 2024, at the national level, China issued the *Action Plan for Strengthening the Care and Protection of Migrant Children*, elevating the care, protection, and developmental support of migrant children to the level of a dedicated national initiative. The plan explicitly calls for the enhancement of care services, the improvement of protective measures, and the promotion of the healthy growth and holistic development of migrant children. However, migrant students may face greater uncertainty and relational vulnerability during the adaptation process, making teacher care a particularly important resource for compensating deficits and buffering stress in their relationship-building (Liu, 2024). At the same time, integration barriers and trust thresholds may make it more difficult for teacher care to translate into stable relationships for migrant children, particularly in contexts of marginalization and limited social support (Zhu et al., 2023). This paradox suggests that the influence of students’ mobility status on the association between teacher care and teacher-student relationships remains unclear and requires further empirical investigation.

Given these insights, this study will examine the mediating role of TSR between teacher care and the SEC of middle school students, and investigate whether this mediation is moderated by migration Status. This study aims to provide a theoretical basis and empirical reference for the cultivation of students’ SEC, thereby supporting their holistic development and future success.

### 1.1 The relationship between teacher care and SEC

Teacher care refers to proactively initiated behavioral patterns by teachers, aimed at establishing stable interpersonal relationships with students. At its core, it involves the recognition and response to students’ emotional and psychological needs (Zhou, 2023). Teacher care is a key factor influencing student development, and its significance has been widely recognized in the international education community (Ye and Cao, 2023). For instance, in the United States, teacher care has been incorporated as a compulsory module of teacher professional development programs (Rogers and Webb, 1991; Elbertson et al., 2025). In China, the Code of Professional Ethics for Primary and Secondary School Teachers explicitly identifies “caring for students” as a fundamental ethical requirement for teachers (Zhang et al., 2024).

Self-Determination Theory emphasizes that individuals are influenced by significant others in their learning environments. When these significant others—such as teachers—display attitudes of encouragement and care, students’ intrinsic motivation is enhanced, leading them to better tap into their internal potential and proactively engage in self-development (He et al., 2025). Empirical studies have shown that teacher care can strengthen the positive impact of students’ SEC on academic engagement (Yin et al., 2023). SEC, also referred to as non-cognitive ability or soft skills (Huang, 2024), is increasingly recognized as a critical dimension of student development. Recent evidence indicates that teacher care significantly influences the cultivation of non-cognitive ability among rural children (Ye and Cao, 2023).

Although current research directly examining the relationship between teacher care and students’ SEC remains limited, relevant insights can be gained by focusing on the dimension of teachers’ emotional support. Emotional support is a key manifestation of teacher care, characterized by attentiveness, empathy, and efforts to build close TSR (Guo et al., 2025). It is a crucial factor in promoting students’ emotional development (Poulou and Denham, 2023). Teachers’ caring behaviors enable students to feel understood, respected, and valued, thereby enhancing their perceived social support. This experience of support not only stimulates students’ motivation to learn (Lavy and Naama-Ghanayim, 2020; Wu and Cai, 2025), but also directly and effectively fosters the development of their SEC (Wang et al., 2025). Therefore, teacher care may be a key factor influencing students’ SEC. Therefore, this study proposes the following hypothesis:

*H1*: There is a positive correlation between teacher care and students’ SEC.

### 1.2 The mediating role of teacher-student relationship

The teacher-student relationship (TSR) is a type of interpersonal relationship that gradually develops between teachers and students through communication and interaction, primarily manifesting in emotional, cognitive, and behavioral communication (Wang et al., 2024a). It has a profound impact on students’ learning and social development (Ria and Eliasa, 2024). Empirical studies have demonstrated a significant positive correlation between teacher care and the quality of TSR (Song et al., 2022). Care theory explains the interactive dynamic between the caregiver and the cared-for (Zhang et al., 2025a). This relationality suggests that teacher care and TSR are interdependent (Derakhshan et al., 2022) further point out that both verbal and non-verbal expressions of care are essential means by which teachers build and maintain high-quality TSR. When students perceive genuine care from their teachers, they tend to experience positive emotional responses and an increased sense of psychological well-being, which in turn promotes the development of positive TSR (Zhou, 2023).

The development of SEC is a process that is continuously enriched and expanded through an individual’s social relationships (Du and Qi, 2023). As one of the most fundamental interpersonal relationships in schools (Ma et al., 2022), a positive TSR provide essential conditions for the development of SEC (Yu, 2023). Students’ perceptions of their TSR are significantly and positively correlated with their SEC (Dietrich et al., 2021; Ma et al., 2026), and can positively predict their levels of SEC (Huang and Zeng, 2023). Positive TSR not only offer emotional support and a sense of security (Saxer et al., 2024), but also help enhance students’ self-awareness and self-concept, thereby strengthening their self-management abilities and reducing the risk of potential problem behaviors (Deng et al., 2025). Such relationships provide a nurturing environment for the development of SEC (Du and Mao, 2018; Deng et al., 2025). Based on the above analysis, teacher care contributes to the establishment of positive TSR, which directly promote the development of SEC. Therefore, this study proposes the following hypothesis:

*H2*: The TSR mediates the relationship between teacher care and students’ SEC.

### 1.3 The moderating role of migration status

According to the Provisional Measures for the Schooling of Migrant Children and Adolescents jointly issued by the Chinese National Education Committee, and Ministry of Public Security (1998), “migrant children” are defined as children aged between 6 and 14 (or 7 and 15) who have temporarily resided in urban areas for over 6 months together with their parents or guardians. Research over the past two decades has shown that, compared to local urban children, migrant children often face marginalization and social exclusion due to their unique living environments and migration experiences (Jiang and Ngai, 2022). During the process of adapting to new environments, they encounter multiple risks (Jiang et al., 2019; Fu et al., 2025), including low family socioeconomic status (Huang and Wang, 2022), strained parent–child relationships (Zhu et al., 2025), peer discrimination (Hamel, 2022), and school adjustment difficulties (Zhu et al., 2022). On the other hand, the parents of migrant children are often compelled by economic necessity to focus on work, leaving them with limited time and energy for communication with their children, which may undermine the development of secure attachment patterns in these children (Tang et al., 2024). Compared to parents of local, migrant parents tend to have higher expectations regarding their children’s academic achievement, hoping their children can change their life circumstances through education. However, more than 40% of migrant parents are unable to provide academic support due to their limited educational background (Gao et al., 2022). This mismatch between high parental expectations and the lack of actual support contributes to increased psychological pressure on migrant children (Li et al., 2024).

Research has shown that teachers play a crucial role in buffering the negative effects of adverse family environments on children (Huang and Zeng, 2023), and can provide a secure emotional foundation for high-risk children (Sigad, 2023). Attachment theory is one of the most prominent theoretical frameworks in the study of TSR (Spilt and Koomen, 2022). This theory posits that, as children grow older, their primary attachment figures shift from caregivers to teachers (Zhang et al., 2025a). Teachers who provide consistent, stable, and reliable emotional support and care can help migrant children with insecure attachment tendencies repair their emotional experiences and foster the development of positive TSR (Wang and Ma, 2025), thereby enabling them to better cope with the challenges posed by migration (Saxer et al., 2024). In contrast, local urban children generally possess more stable and diverse social support systems, of which teachers are merely one source. In this context, a single encouraging word or caring gesture from a teacher may have a far greater emotional impact and positive effect on migrant children than on their local non-migrant children, thereby more effectively enhancing TSR (Zhang and Feng, 2025). Specifically, compared to local non-migrant children, the TSR among migrant children is more susceptible to the positive influence of increased teacher care. Based on the aforementioned findings, this study proposes the following hypothesis:

*H3*: Migration status moderates the relationship between teacher care and TSR.

**Figure 1:**
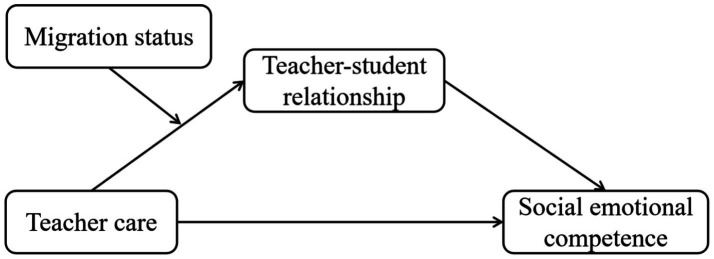
Schematic diagram of model (source: constructed based on a systematic review and synthesis of existing literature related to the core variables).

Based on the literature review and research hypotheses mentioned above, this study constructs a moderated mediation model (as shown in Figure 1).

## 2 Method

### 2.1 Participants

This study utilizes data from the China Education Panel Survey (CEPS), a nationally representative, multi-level foundational dataset designed and implemented by the China Survey and Data Center at Renmin University of China. To date, two waves of data have been released: 2013–2014 and 2014–2015. A total of 112 junior secondary schools were randomly selected from 28 county-level administrative units across China, encompassing 438 classes in Grade 7 and Grade 9. The CEPS survey instruments were tailored to different target groups, including students, parents, homeroom teachers, subject teachers, and school administrators. With the exception of parents, all other respondents were required to complete the questionnaires collectively on-site. This study adopts the 2013–2014 cross-sectional data for preliminary analysis. The dataset includes information on students’ basic demographic characteristics, teacher care, TSR, and students’ SEC, after removing cases with critical missing values, the final effective sample size retained for analysis was 12,319 students.

### 2.2 Measures

#### 2.2.1 Teacher care

Based on previous studies, this research measured the level of teacher care using two items from the CEPS parent questionnaire: “Do you think the teacher is responsible for this child?” with response options ranging from “1 = Not responsible at all” to “5 = Very responsible”; and “Do you think the teacher has patience with this child?” with response options ranging from “1 = Not patient at all” to “5 = Very patient” (Zhang et al., 2025a). Both items were rated on a five-point Likert scale. The average score of the two items was calculated, with higher scores indicating a higher level of teacher care. The Cronbach’s *α* coefficient of the scale in this study was 0.82.

#### 2.2.2 TSR

The TSR was measured using two items from the CEPS parent questionnaire: “Does the child like their homeroom teacher” and “Does the child like other teachers” (Zhang et al., 2025a). Both items were rated on a four-point Likert scale: “1 = Not at all,” “2 = Not very much,” “3 = Fairly,” and “4 = Very much.” The average score of the two items was calculated, with higher scores indicating a better TSR. The Cronbach’s *α* coefficient of the scale in this study was 0.77.

#### 2.2.3 SEC

This study measured students’ SEC with reference to the research by Gong et al. (2024), drawing on the framework developed by the Collaborative for Academic, Social, and Emotional Learning (CASEL), which includes five core components: self-awareness, self-management, social awareness, relationship skills, and responsible decision-making. Twelve items from the CEPS student questionnaire were used for this measurement, such as “Do you have confidence in your future?,” “I often participate in school or class-organized activities.” Four items under the self-management dimension were reverse-coded. Higher total scores across all items indicated stronger SEC. The Cronbach’s *α* coefficient of the scale in this study was 0.80.

#### 2.2.4 Migration status

According to the CEPS data user manual, students’ migration status was determined based on the item “What is your current household registration location?” in the student questionnaire. The response options include “Local non-migrant,” “Intra-provincial migrant,” and “Inter-provincial migrant.” In this study, children who chose either intra-provincial or inter-provincial migrants were grouped together and classified as migrant children.

#### 2.2.5 Control variables

Control variables include individual-level and family-level factors that may influence students’ SEC. These variables comprise gender, age, only-child status, parental education level, parental relationship quality, and family economic condition. Details of each variable are presented in Table 1.

**Table 1:** Description of variables in statistical analysis.

Variable names	Item		Definition
Gender	What is your gender?		1-male, 0-female
Age	What is your age?		Number of years
Only child	Are you an only child?		1-Only child, 2-Non-only child
Family economic condition	How is your family’s current financial situation?		1-Poor, 2-Middle class, 3-Rich
Parents’ highest level of education	What is the educational level of your father/mother?		1-No education whatsoever, 2-Elementary school, 3-Junior high school, 4-Junior college/technical school, 5-vocational high school, 6-High school, 7-College, 8-Undergraduate, 9-Graduate and above.
Relationship between parents	My parents have a good relationship with each other.		1-No, 2-Yes
Teacher care	Do you think the teacher is responsible for this child? Do you think the teacher has patience with this child?		A 5-point Likert scale:1 (Not at all) to 5 (Very)
TSR	Does the child like their homeroom teacher? Does the child like other teachers?		A 4-point Likert scale:1 (strongly unlike) to 4 (strongly like)
SEC	Self-awareness	Do you have confidence in your future?	A 4-point Likert scale: 1 (strongly unconfident) to 4 (strongly confident)
	Self-management	In the past 7 days, have you been feeling frustrated/depressed/unhappy/the life is meaningless/sadness?	A 5-point Likert scale: 1 (never) to 5 (always)
	Social awareness	I often participate in activities organized by the school or classroom. I think I’m easy to get along with.	A 4-point Likert scale: 1 (strongly disagree) to 4 (strongly agree)
	Relationship skills	Most of my classmates are friendly to me. I feel close to the people at this school.	A 4-point Likert scale: 1 (strongly disagree) to 4 (strongly agree)
	Responsible decision-making	Even if I’m a little under the weather, or have another reason to stay home, I still try to go to school. Even if it’s homework I do not like, I’ll do my best to do it. Even if the homework takes forever to do, I still keep trying my best to do it.	A 4-point Likert scale: 1 (strongly disagree) to 4 (strongly agree)
Migration status	What is your current household registration location?		1-Non-migrant, 2-Migrant

### 2.3 Data analysis

This study used SPSS 28.0 and PROCESS macro for data analysis. The analytical procedure was as follows: First, common method bias was tested, followed by descriptive statistics and correlation analysis to gain a basic understanding of the variables and their interrelationships. Second, based on the research objectives and model hypotheses, Model 8 of the PROCESS macro was employed to examine the moderating effect. A bootstrap method with 5,000 resamples was used to estimate the 95% confidence intervals for the moderation analysis.

## 3 Results

### 3.1 Descriptive statistics

The descriptive statistics of the research sample are presented in Table 2. Among the sample, 17.45% are migrant children. The proportion of male students is 50.7%, while female students account for 49.3%. The average age is 13.87 years. Only children constitute 46.90% of the sample. Most families are in good economic condition. Approximately 38.95% of parents have attained a highest educational level of senior high school or above. About 83.48% of parents report having a good relationship with each other.

**Table 2:** Descriptive statistics.

Variable type	Variable	*M*	SD	Min	Max
Independent variable	Teacher care	4.44	0.58	1	5
Mediating variable	TSR	3.44	0.49	1	4
Dependent variable	SEC	3.36	0.46	1.20	4.20
Moderating variable	Migration status	1.19	0.39	1	2
Control variable	Gender	1.51	050	1	2
	Age	13.87	1.23	11	17
	Only child	1.53	0.50	1	2
	Family economic condition	1.88	0.48	1	3
	Parents’ highest level of education	4.65	2.05	1	9
	Relationship between parents	1.85	0.36	1	2

### 3.2 Common method bias test

As all data in this study were obtained through self-reports, the results may be subject to common method bias. To assess the presence of common method bias, Harman’s single-factor test was conducted. The results indicated that four factors had eigenvalues greater than 1. The first factor accounted for 23.56% of the total variance, which is below the critical threshold of 40%, suggesting that there is no serious common method bias (Zhou and Long, 2004; Zhang et al., 2025b).

### 3.3 Correlation analysis

Correlation analyses were performed on migration status, teacher care, TSR, and SEC. The results showed that both teacher care and TSR were significantly positively correlated with SEC (*p* < 0.01). Migration status was significantly negatively correlated with teacher care, TSR, and SEC (*p* < 0.01). Detailed results are presented in Table 3.

**Table 3:** Correlation analysis.

Variables	1	2	3	4
Migration status	1			
Teacher care	−0.09^**^	1		
TSR	−0.08^**^	0.47^**^	1	
SEC	−0.06^**^	0.23^**^	0.28^**^	1

*p < 0.05, **p < 0.01, ***p < 0.001.

### 3.4 Mediating effect analysis

First, all variables were standardized. After controlling for gender, age, only-child status, parental education level, parental relationship quality, and family economic condition, the mediation model was tested using the SPSS macro PROCESS (Model 4). As shown in Table 4, teacher care significantly and positively predicted SEC (*β* = 0.19, *p* < 0.001). When the mediator variable—TSR—was included, teacher care still significantly predicted SEC (*β* = 0.11, *p* < 0.001). Additionally, teacher care positively predicted the TSR (*β* = 0.46, *p* < 0.001), and TSR positively predicted SEC (*β* = 0.19, *p* < 0.001). These results indicate that TSR mediates the association between teacher care and adolescents’ SEC.

**Table 4:** Mediation regression analysis results.

Result variable	Prediction variable	*R*	*R* ^2^	*β*	*t*	95%CI
SEC		0.33	0.11^***^			
	Teacher care			0.19	22.47^***^	[0.18,0.21]
TSR		0.48	0.23^***^			
	Teacher care			0.46	57.01^***^	[0.44,0.47]
SEC		0.37	0.13^***^			
	Teacher care			0.11	11.02^***^	[0.09,0.12]
	TSR			0.19	20.35^***^	[0.18,0.21]

### 3.5 Moderated mediation model test

To examine whether migration status moderates the relationship between teacher care and TSR, this study utilized the SPSS macro PROCESS (Model 8) for data analysis. Gender, only-child status, parental education level, parental relationship quality, and family economic condition were included as control variables. As shown in Table 5, teacher care significantly and positively predicted TSR (*β* = 0.44, *p* < 0.001). Moreover, the interaction term between teacher care and migration status also positively predicted TSR (*β* = 0.05, *p* < 0.001), indicating that migration status moderates the relationship between teacher care and TSR.

**Table 5:** Analysis of moderated mediation effects.

Regression equation		Fitting index		Standardized coefficients		
Result variable	Prediction variable	*R* ^2^	*F*	*β*	*t*	95%CI
TSR		0.24	425.25^***^			
	Teacher care			0.44	54.67^***^	[0.43, 0.46]
	Migration status			−0.03	−3.41^***^	[−0.04, −0.01]
	Teacher care × Migration status			0.05	6.78^***^	[0.03, 0.06]
SEC		0.13	239.81^***^			
	Teacher care			0.11	11.02^***^	[0.09, 0.12]
	TSR			0.19	20.35^***^	[0.18, 0.21]

**Figure 2:**
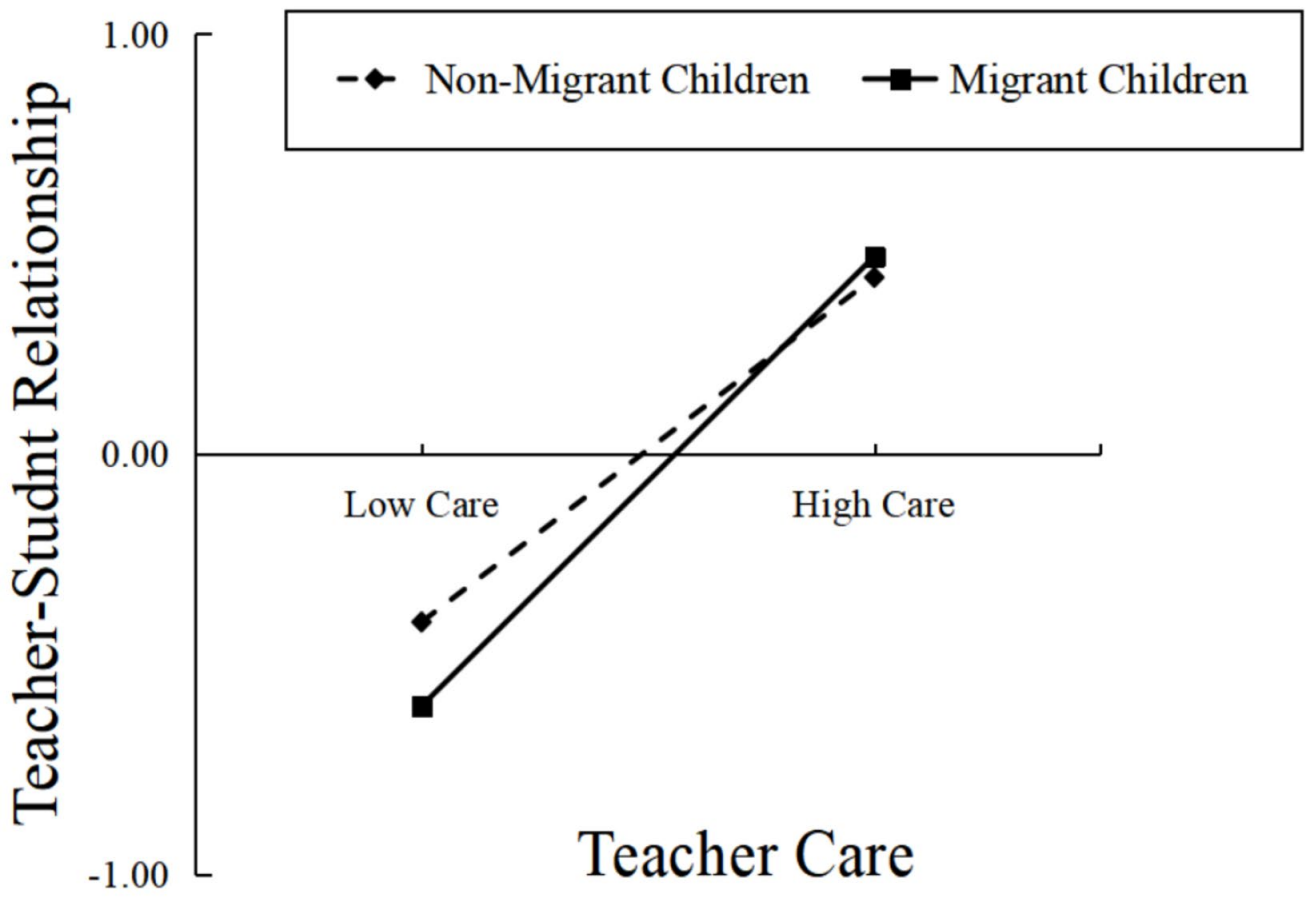
Moderation effect of migration status on teacher care and TSR (source: prepared based on the analysis results of the 2013–2014 CEPS data).

To further examine how migration status moderates the relationship between teacher care and TSR, a simple slopes analysis was conducted. As shown in Figure 2, the results revealed that the predictive effect of teacher care on TSR was stronger among migrant children (*β* = 0.54, *t* = 35.01, *p* < 0.001), whereas the effect was weaker among non-migrant children (*β* = 0.42, *t* = 45.36, *p* < 0.001).

**Figure 3:**
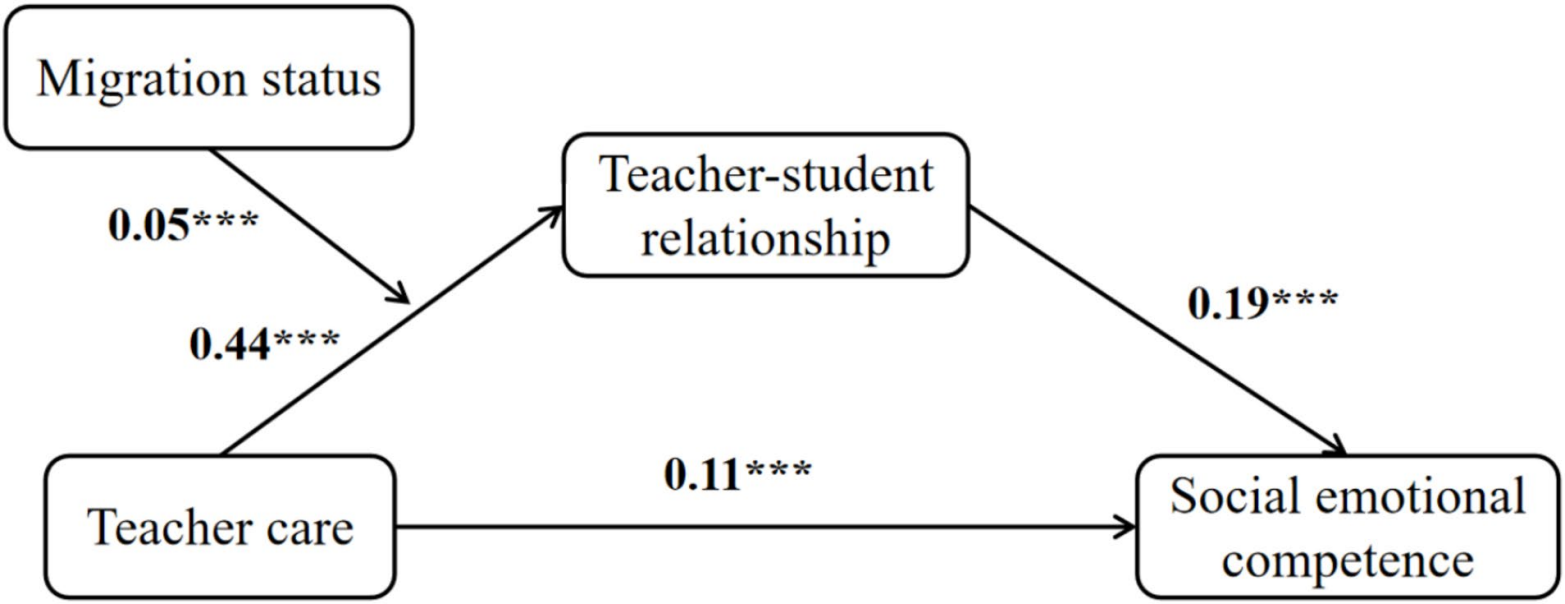
A moderated mediation model (source: constructed based on the analysis results of the 2013–2014 CEPS data).

The above results indicate that teacher care, TSR, student migration status and SEC together form a moderated mediation model. Specifically, TSR mediates the association between teacher care and students’ SEC, while migration status moderates the relationship between teacher care and TSR. Thus, the proposed moderated mediation model is supported (Figure 3).

## 4 Discussion

This study investigates the relationship between teacher care and adolescents’ SEC, as well as the underlying mechanisms involved. The results indicate that the TSR mediates the association between teacher care and SEC. Moreover, the path from teacher care to TSR is moderated by students’ migration status. These findings provide evidence to understand the mechanisms influencing adolescents’ SEC and have important implications for enhancing adolescents’ SEC and mental health.

### 4.1 The relationship between teacher care and SEC

The results of this study indicate a significant positive correlation between teacher care and students’ SEC, confirming Hypothesis 1. In other words, the more caring behaviors teachers exhibit, the higher the level of social emotional development. This finding aligns with previous research, teacher care helps students establish a sense of belonging and emotional connection within the educational environment (Miller and Gkonou, 2023). This finding is also supported from the perspective of Self-Determination Theory. As key supportive figures in children’s development, the importance and influence of teachers have been emphasized in numerous studies (Wang et al., 2025). As children enter school, teachers gradually become critical agents in their socialization and emotional development. Through daily interactions and guidance, teachers create a safe, supportive, and encouraging learning environment, which in turn helps students build self-confidence, learn to cooperate, develop empathy, and acquire strong social skills (Kusumaningsih and Sun, 2025). When teachers treat every student equally, and respect individual differences and uniqueness, students experience feelings of being valued and recognized (Zheng, 2022). As a result, students task engagement, adaptability, and ability to cope with environmental challenges are significantly enhanced (Prananto et al., 2025), which contributes to the formation of positive self-perception and the development of stronger SEC. Therefore, teacher care is an indispensable component in the development of students’ SEC. It fosters students’ holistic growth in emotional, social, and moral domains.

### 4.2 The mediating role of TSR

This study confirms the mediating role of the TSR between teacher care and students’ SEC, thereby supporting Research Hypothesis 2. That is, teacher care indirectly promotes the development of students’ SEC by enhancing the quality of TSR. Teacher care is a primary form of teacher-student interaction (Zhang et al., 2025a) and a crucial factor in building high-quality TSR (Jiang et al., 2024). This result supports Noddings (2012) theory of care, teachers’ emotional support and care for students foster the development of harmonious TSR, thereby improving students’ psychological well-being and overall life satisfaction (Saxer et al., 2024). Teacher care enhances students’ willingness to engage in open interpersonal exchanges, express their emotions authentically, and share personal and academic experiences with teachers (Shen et al., 2024). These processes collectively strengthen the TSR, transforming it from a traditionally institutional, task-oriented relationship into a meaningful human connection infused with emotional reciprocity and educational significance (Wang et al., 2024b). Notably, a high-quality TSR acts as a proximal developmental context that further promotes the acquisition and refinement of students’ SEC, as it provides a safe environment for practicing social skills (e.g., empathy, conflict resolution) and regulating emotions (Du and Qi, 2023). In summary, when teachers demonstrate equitable, non-discriminatory attitudes toward all students—ensuring that each individual feels valued, cared for, and respected—it lays the foundation for the establishment of a secure, attachment-based TSR (Yang et al., 2025). This secure relational attachment serves as a buffer against psychological distress and provides students with a sense of emotional safety, which is a prerequisite for the optimal development of SEC (Yuan et al., 2021; Di Lisio et al., 2025).

### 4.3 The moderating role of migration status

This study confirms that students’ migration status moderates the relationship between teacher care and TSR, thereby supporting Research Hypothesis 3. Specifically, teacher care exerts a stronger positive influence on TSR among migrant children than on their local non-migrant peers. This can be attributed to the fact that, due to family migration and challenges in social integration, migrant children often lack stable emotional support (Liu, 2023). This lack makes them more sensitive to teacher care and more likely to perceive it as a powerful source of external support (Wang and Ma, 2025). This emotional compensation mechanism can effectively mitigate their emotional deprivation, allowing them to feel valued and loved. These positive feelings, in turn, increase their willingness to form close bonds with teachers and foster the development of high-quality TSR.

On the other hand, migrant children generally come from families with lower socioeconomic status and have limited access to educational resources, which often results in poor learning habits and academic performance (Li and Su, 2025). When teachers offer continuous attention, thoughtful care, and academic encouragement, migrant children are more likely to view teachers as significant sources of psychological support. This helps students build trust in their teachers and develop positive emotional experiences related to learning (Wang, 2025). As a result, they are more willing to participate in classroom interactions, complete learning tasks actively, and make greater efforts to reciprocate the teacher’s care, thus facilitating the formation of positive TSR (Lavy and Naama-Ghanayim, 2020; Wang and Ma., 2025).

### 4.4 Limitations and perspectives

First, some of the data in this study were obtained from parent-report questionnaires, which may carry the risk of reporting bias because caregivers’ perceptions can be influenced by social desirability, subjective interpretation, and informant discrepancies in reporting youth behavior (Martinez-Yarza et al., 2024). Future research could consider incorporating multi-perspective data, such as self-reports from students, teacher reports, and parent evaluations, as well as classroom observations to provide a more comprehensive and objective understanding of the true relationship between the variables.

Second, adolescents’ SEC is jointly shaped by the family, peer, and school relational systems (Yang et al., 2025). However, the present study primarily focuses on the influence mechanisms of teachers and does not incorporate other important interpersonal relationships. Therefore, future research should further examine the joint and interactive effects of multiple relational systems on adolescents’ SEC.

Third, research has suggested that there is a bidirectional predictive relationship between teacher care and TSR (Zhang et al., 2025a), and that the development of SEC is a continuously evolving and complex dynamic system. However, the cross-sectional design used in this study is insufficient to disentangle reciprocal interactions and developmental processes (Savitz and Wellenius, 2023). Future research could employ longitudinal designs with multiple time points to more accurately uncover the causal pathways and dynamic mechanisms between teacher care and students’ SEC.

Fourth, migrant children account for only 17.45% of the total sample in this study. Although the moderation effect analysis yielded statistically significant results, the weak effect sizes may indicate limited practical significance and could be influenced by the relatively small proportion of migrant students, which also constrains the generalizability and reliability of the findings (Edelsbrunner and Thurn, 2024). Future research may conduct targeted investigations in schools with a high proportion of migrant students, expand the sample size of migrant students, and thereby improve the statistical power of the analysis.

Finally, this study utilizes CEPS data collected in 2013–2014. As the data are drawn from a national large-scale sample survey, the research findings possess strong generalizability and representativeness. Yet a decade has passed since the data collection, and with the advancement of educational informatization, the deepening of curriculum reform, and shifts in the developmental contexts of adolescents, the patterns of teacher-student interaction and the contexts of students’ social–emotional development may have changed accordingly (Wang C. et al., 2024; Wang et al., 2024a,b). As such, the applicability of this study’s conclusions to the current educational context may be somewhat limited. Future research may adopt updated waves of the survey data or other recent empirical survey datasets to replicate and validate the research model of this study, thereby enhancing the temporal adaptability of the conclusions.

### 4.5 Educational suggestion

At the school level, it is essential to build a supportive environment and improve the institutional guarantees for teacher care (Zhang and He, 2024). First, a teacher evaluation and incentive mechanism that integrates a care-oriented approach should be established. Teachers’ emotional support and caring behaviors toward students should be incorporated into the teacher assessment system, motivating teachers to actively practice caring education and make care a conscious and intentional part of their educational behavior. Second, systematic teacher training should be implemented to enhance emotional education competence. By regularly organizing professional development programs focused on social emotional learning, positive psychology, and teacher-student communication skills, teachers can be equipped with strategies for recognizing students’ emotions and building positive TSR, thereby strengthening their capacity to provide emotional support (Masry-Herzallah, 2025).

At the teacher level, it is essential to foster high-quality TSR and leverage the guiding role of emotions. First, establishing positive TSR is a critical component of teaching practice (Li et al., 2022). Teachers should integrate the concept of care into their daily teaching activities and employ diverse caring behaviors to meet students’ emotional needs, thereby laying a solid foundation for the development of their SEC. Second, teachers should recognize and respect individual differences among students and implement differentiated instruction accordingly. For vulnerable groups requiring special attention—such as migrant children (Wang and Ma, 2025), left-behind children (Mo et al., 2023), and children living in poverty (Söngüt and Gözübüyük, 2025)—teachers should provide increased care and support. By establishing stable and supportive TSR, teachers can help mitigate the potential risks associated with these students’ disadvantaged circumstances, thus effectively promoting the development of their SEC.

## 5 Conclusion

This study was designed to address the overarching research question: What is the mechanism through which teacher care influences middle school students’ social–emotional competence (SEC), and does this mechanism differ between migrant and local non-migrant students? To answer this, we tested three core hypotheses: Hypothesis 1: Teacher care would be positively associated with students’ SEC. Hypothesis 2: Teacher-student relationship (TSR) would mediate the positive relationship between teacher care and students’ SEC. Hypothesis 3: Students’ migration status would moderate the relationship between teacher care and TSR.

This study, drawing on nationally representative educational data, was the first to empirically examine the relationship between teacher care and students’ SEC. The results indicated a significant positive correlation among teacher care, TSR, and students’ SEC, which fully corroborated Hypothesis 1. Moderated mediation model analysis revealed that TSR partially mediated the relationship between teacher care and students’ SEC, providing support for Hypothesis 2. While students’ migration status moderated the relationship between teacher care and TSR. Further simple slope analysis showed that, when receiving teacher care, migrant children reported significantly higher-quality TSR compared to their local non-migrant peers, which fully corroborated Hypothesis 3.

## Data Availability

The raw data supporting the conclusions of this article will be made available by the authors, without undue reservation.
